# Phospholamban R14del disease: The past, the present and the future

**DOI:** 10.3389/fcvm.2023.1162205

**Published:** 2023-04-18

**Authors:** Elizabeth Vafiadaki, Pieter C. Glijnis, Pieter A. Doevendans, Evangelia G. Kranias, Despina Sanoudou

**Affiliations:** ^1^ Center of Basic Research, Biomedical Research Foundation of the Academy of Athens, Athens, Greece; ^2^ Stichting Genetische Hartspierziekte PLN, Phospholamban Foundation, Wieringerwerf, Netherlands; ^3^ Netherlands Heart Institute, Utrecht, Netherlands; ^4^ Department of Cardiology, University Medical Center Utrecht, Utrecht, Netherlands; ^5^ Department of Pharmacology and Systems Physiology, University of Cincinnati College of Medicine, Cincinnati, OH, United States; ^6^ Clinical Genomics and Pharmacogenomics Unit, 4th Department of Internal Medicine, Attikon Hospital, Medical School, National and Kapodistrian University of Athens, Athens, Greece; ^7^ Center for New Biotechnologies and Precision Medicine, Medical School, National and Kapodistrian University of Athens, Athens, Greece

**Keywords:** phospholamban, R14del mutation, arrhythmias, cardiomyopathy, precision medicine, therapy

## Abstract

Arrhythmogenic cardiomyopathy affects significant number of patients worldwide and is characterized by life-threatening ventricular arrhythmias and sudden cardiac death. Mutations in multiple genes with diverse functions have been reported to date including phospholamban (PLN), a key regulator of sarcoplasmic reticulum (SR) Ca^2+^ homeostasis and cardiac contractility. The PLN-R14del variant in specific is recognized as the cause in an increasing number of patients worldwide, and extensive investigations have enabled rapid advances towards the delineation of PLN-R14del disease pathogenesis and discovery of an effective treatment. We provide a critical overview of current knowledge on PLN-R14del disease pathophysiology, including clinical, animal model, cellular and biochemical studies, as well as diverse therapeutic approaches that are being pursued. The milestones achieved in <20 years, since the discovery of the PLN R14del mutation (2006), serve as a paradigm of international scientific collaboration and patient involvement towards finding a cure.

## Introduction

Arrhythmogenic cardiomyopathy (ACM), a genetic disease with a prevalence of approximately 1:5,000, is characterized by life-threatening ventricular arrhythmias and sudden cardiac death (SCD) in apparently healthy young adults (1, 2). SCD accounts for high mortality rates worldwide and represents a major challenge for healthcare systems. Arrhythmias are difficult to predict and treat. Currently available therapeutic approaches target primarily the symptoms, have limited success and do not prevent disease progression (2, 3). This is partly due to the polygenic nature of ACM. Although ACM was originally associated with mutations predominantly in desmosomal genes, over the years landmark discoveries in this field unveiled pathogenic mutations in genes with highly diverse biological roles (1, 2, 4). Among them, the identification of pathogenic phospholamban (PLN) variants, opened the way to a fascinating journey of scientific discoveries that are critically reviewed herein (5).

PLN is a 52 amino acid transmembrane protein that modulates Ca^2+^ homeostasis and cardiac contractility through reversible inhibition of the sarcoplasmic reticulum (SR) calcium ATPase (SERCA2a) activity. Specifically, PLN acts by lowering the affinity of SERCA2a for Ca^2+^ and this inhibitory effect on SERCA2a is relieved by phosphorylation (6). PLN is phosphorylated at residue serine 16 (Ser-16) by the cAMP-dependent protein kinase (PKA) and at threonine 17 (Thr-17) by the Ca^2+^-calmodulin-dependent protein kinase (CaMKII). In its dephosphorylated state, PLN interacts with SERCA2a and inhibits its affinity for Ca^2+^, however, upon *β*-adrenergic stimulation, phosphorylation of PLN relieves this inhibitory effect on SERCA2a, leading to enhanced SR Ca^2+^ transport and cardiac relaxation (6). In addition to phosphorylation, cytosolic Ca^2+^ concentration is also known to affect the PLN inhibitory effect on SERCA2a. In particular, at low Ca^2+^ concentration, PLN interacts and reversibly inhibits SERCA2a affinity for Ca^2+^, while elevations in Ca^2+^ concentration lead to dissociation of the SERCA2a/PLN protein complex and SERCA2a inhibition relief that consequently leads to cardiac relaxation (7).

The central role of PLN in cardiac function is highlighted both by the multiple and multifunctional binding partners (8–13), but also importantly, by the detrimental effects of PLN mutations (14, 15). *PLN* pathogenic variants have been reported to date in ACM, dilated cardiomyopathy (DCM) and heart failure patients, including missense (c.25C > T (p.Arg9Cys), c.26G > A (p.Arg9His), c.73C > T (p.Arg25Cys)), nonsense (c.116T > G (p.Leu39Ter), c.4G > T, (p.Glu2Ter)), deletion (c.40_42delAGA, (p.Arg14del) and promoter (−36 A > C, −42 C > G) genetic variants (5, 16–22). Among these, the deletion of arginine at amino acid residue 14 (R14del) has lately attracted considerable attention.

**Figure 1:**
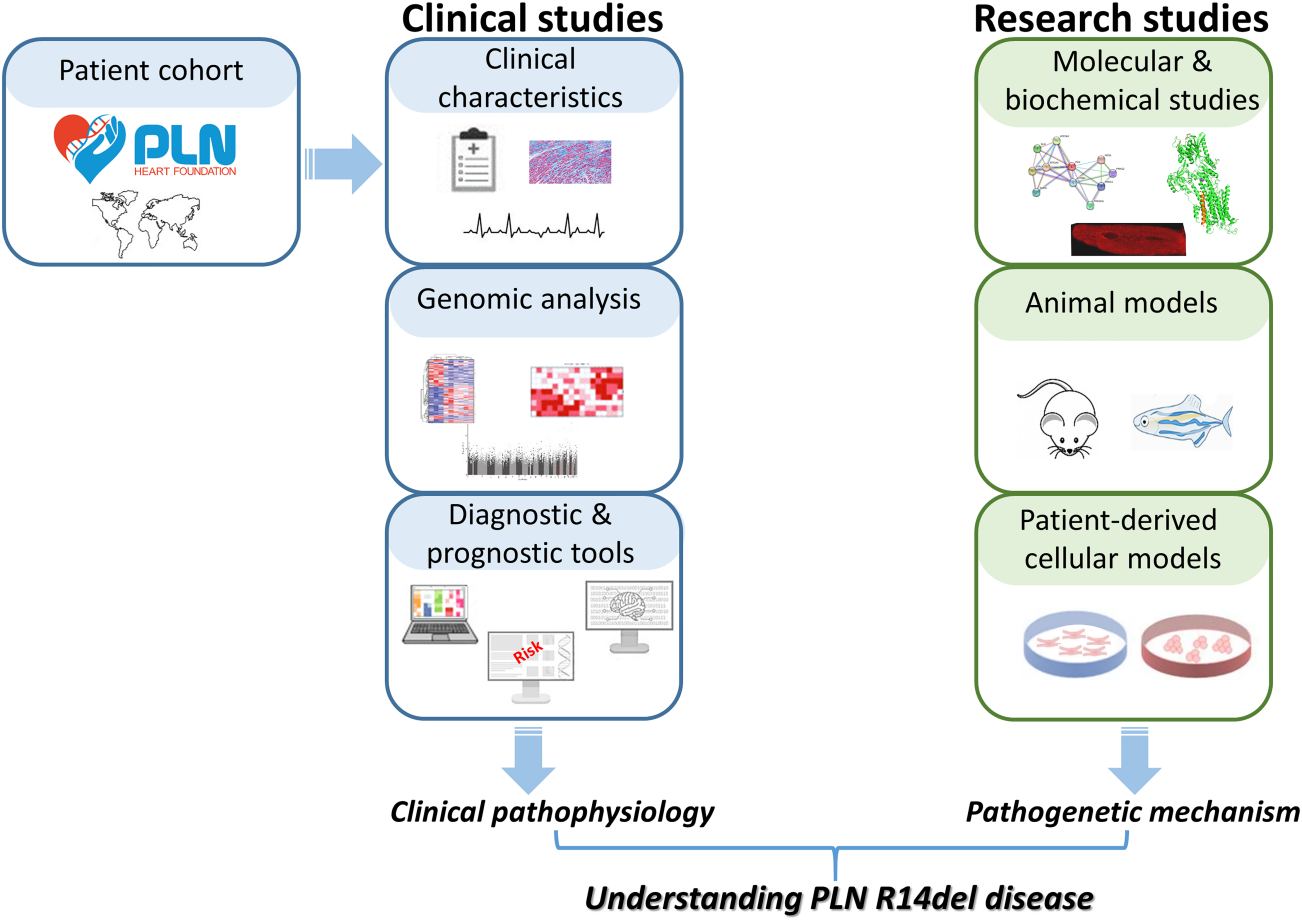
Approaches employed towards deciphering the pathophysiology of PLN-R14del disease. With the contribution of the PLN Foundation, patient communities worldwide are coming together to enable a multitude of clinical studies which have helped significantly in better defining the clinical characteristics of PLN-R14del disease. In addition to this, research strategies aiming at the biochemical, molecular, cellular and animal model levels are being explored in order to decipher the pathogenetic mechanisms underlying PLN-R14del. Collectively, the increasing number of clinical and research studies have significantly enhanced our understanding of PLN-R14del disease.

The PLN-R14del mutation was originally identified by Professor Kranias' team in a large Greek family pedigree with DCM and symptoms attributed to arrhythmias (5, 23). Subsequently, it has been reported in an increasing number of patients worldwide, including multiple European populations but also in the United States, Canada, Japan and China (24–27). Strikingly, in the Netherlands the PLN-R14del genetic variant is the most prevalent cardiomyopathy-related mutation (>1,500 carriers), present in ∼12% of patients with ACM and ∼15% of patients with DCM (27–29). The Dutch PLN-R14del patients have established the PLN Foundation to fund and promote scientific research towards finding a cure while raising awareness on PLN-R14del disease, as well as supporting patients and their families (Figure 1) (30). In addition, a transatlantic network of excellence consortium was established, funded by the Leducq Foundation, which focuses on deciphering the pathophysiology of PLN-R14del disease to facilitate the development of targeted therapeutic interventions for the patients (31). Valuable resources for these studies are cardiac tissue banks, such as the recently established Netherlands Heart Tissue Bank (Hartenbank; https://www.hartenbank.nl/en/), which store and make freely available to the research community high-quality cardiac tissue along with detailed medical data from deceased individuals with or without heart disease (32). The present review is the first to provide a comprehensive overview of the considerable scientific progress achieved since the discovery of PLN R14del in 2006. In particular, we summarize current knowledge on PLN-R14del pathophysiology, with emphasis on research evidence from animal, cellular and biochemical studies that have all contributed in delineating key pathways that underlie disease pathogenesis in PLN-R14del hearts (Figure 1).

## Clinical characteristics of PLN-R14del patients

Human carriers of the PLN-R14del variant exhibit highly variable phenotypes, ranging from asymptomatic to cardiomyopathic with clinical features of both ACM and DCM that may progress to heart failure and SCD (28). The underlying etiology of this highly variable expressivity remains unclear, while the implication of modifier genes is a possibility, similar to other PLN variants (33). PLN-R14del patients exhibit low-voltage electrocardiograms (ECGs), negative T waves in left precordial leads, high prevalence of malignant ventricular arrhythmias and end-stage heart failure (27, 29). Sex-specific differences in low-voltage ECGs have been observed, with their occurrence being more common in females but having greater predictive value in males, who experience higher incidence of sustained ventricular arrhythmias (34). A risk prediction model of ventricular arrhythmias has been proposed and its application is expected to help improve individual risk prediction and accuracy in decision making for primary prevention (35). Disease penetrance is incomplete and age-dependent, with symptoms developing more often within the fifth decade (27, 36). However, patients have poor prognosis and exhibit high mortality due to SCD even at younger age indicating the importance of early diagnosis (27, 28). Towards this, efforts are now being made on identifying early cardiac abnormalities that precede symptoms development (37). In addition, patient diagnosis will be aided by the development of automatic tools based on artificial intelligence in order to enable efficient patient screening (38–40). Histological features observed in PLN-R14del patients' hearts include extensive fibrosis and fibrofatty replacement (41, 42), with cardiac fibrosis appearing as an early feature of the disease that often occurs in presymptomatic carriers before the onset of overt disease (43). Exploration of the prognostic value of fibrosis biomarkers has revealed moderate correlation to PLN-R14del clinical parameters and disease severity (44). Concomitantly, a prospective randomized controlled clinical trial (PHOspholamban RElated CArdiomyopathy intervention Study, iPHORECAST) is currently evaluating the efficacy of the antifibrotic agent eplerenone in the prevention or delay in progression of disease (www.clinicaltrials.gov, register number NCT01857856) (45). Accumulation of lipid droplets, impaired fatty acid oxidation and mitochondrial integrity disruption have also been observed in PLN-R14del patient hearts (46). Another characteristic of PLN-R14del disease is the presence of aggregates that are mainly observed in the perinuclear region (47, 48). These PLN-containing aggregates appear to be specific for PLN-R14del patients as they were not observed in the hearts of other DCM or ACM patients, suggesting their diagnostic value (47, 48). Detailed immunohistochemical analysis of these aggregates revealed the presence of autophagy-related marker proteins such as p62 (sequestosome-1) and microtubule-associated protein light chain 3 (LC3) along with PLN, therefore indicating impaired autophagic protein degradation in PLN-R14del hearts (48).

Currently, there are no specific treatments for PLN-R14del patients, who at some point will require implantation of a cardiodefibrillator, or left ventricular assist device, or may eventually be in need of cardiac transplantation (28, 31, 49). Given the ever increasing number of detected PLN-R14del carriers worldwide, which is most likely attributed to increased awareness and accurate diagnosis, it has now become evident that understanding PLN-R14del pathophysiology (Figure 1) is essential in order to address the urgent need for therapeutic strategies aiming towards precision medicine for PLN-R14del patients.

## Animal models of PLN-R14del

The generation of animal models for PLN-R14del has provided critical insights into the pathophysiological mechanisms underlying this human mutation (Table 1). Initially, the impact of PLN-R14del in the heart was assessed in a cardiac specific overexpression mouse model (5). These mice presented with pathological features that recapitulated human disease such as ventricular dilatation, myocyte disarray and myocardial fibrosis, and exhibited high rates of mortality, with the majority of the mice dying within 16 weeks of age (5). Ca^2+^ uptake measurements in hearts from overexpressing mice determined enhanced inhibition of the Ca^2+^ affinity of SERCA2a, however this is based on a limited number of mice due to increased mortality issues. Importantly, this inhibitory effect was not fully relieved by PKA phosphorylation, suggesting chronic suppression of SERCA2a (5). PLN-R14del was initially shown to be phosphorylated by PKA at similar levels to PLN-WT (5), however this has been challenged by subsequent studies which have reported lack of PLN-R14del phosphorylation at the Ser-16 site (59–62). This could explain the failure by PLN-R14del to relieve inhibitory effects on SERCA2a upon phosphorylation and suggests that suppression of lusitropy could contribute to disease pathology, especially under stress conditions. Similar lusitropic effects have also been described for another *PLN* mutation, namely PLN-R9C, that in heterozygote state exerts a dominant-negative effect on PLN-WT by impairing protein phosphorylation, causing chronic inhibition of SERCA2a and blunted *β*-adrenergic stimulation response (21, 63, 64). Based on the fact that loss of lusitropy has been associated with a range of DCM-associated mutations, strategies towards its restoration could have promising therapeutic potential (65).

**Table 1:** PLN-R14del animal models and key phenotypic features.

Animal model	Age studied	Animal studied	Phenotype	Main molecular alterations	Reference
Overexpression m*Pln*-*R14del* mouse model	6 weeks	Heterozygotes	Ventricular dilation, myocardial fibrosis, early mortality (2–16 weeks)	Increased inhibition of SERCA2a	(5)
Overexpression m*Pln*-*R14del* in PLN-KO mouse model	8–13 weeks	Homozygotes	Cardiac hypertrophy, ventricular dilation, myocardial fibrosis	Increased contractility, and Ca^2+^ transients, mislocalization to plasma membrane	(50)
Knock-in m*Pln*-*R14del* mouse model	12-, 18- and 20 months	Heterozygotes	Cardiac fibrosis, contractile dysfunction, increased propensity to arrhythmias (at 3 months)	Increased expression of fibrotic genes, PLN aggregates	(51)
	3–8 weeks	Homozygotes	Cardiac fibrosis and dilatation, contractile dysfunction, increased propensity to arrhythmias, early mortality (within 2 months)	Altered expression of protein homeostasis systems, PLN aggregates.	(51, 52)
Knock-in h*PLN*-*R14del* mouse model	12–14 weeks	Heterozygotes	Bi-ventricular dilatation, increased propensity to arrhythmias	Depressed contractile parameters and Ca^2+^ kinetics, prolongation of action potential duration, increased propensity to arrhythmias	(53–57)
	12 months	Heterozygotes	Normal cardiac function and geometry, increased fibrosis and lipid droplets	Depressed contractile parameters and Ca^2+^ kinetics, structural abnormalities in Z-disc formation or organization	(55)
Knock-in *plna-R14del* zebrafish model	10 months and 2 years	Homozygotes	Increased cardiac size, contractility defects, cardiac remodelling	Contractility defects, impaired Ca^2+^ kinetics,increased action potential alternans	(58)

Since the original report, additional models have been created using different experimental strategies. This includes the generation of a mouse model that expresses the human PLN-R14del mutant in the PLN null (PLN-KO) mouse heart (50). This particular model enabled the study of the pathological effects of the mutation in the absence of endogenous PLN. Detailed analysis of this model indicated a hyperdynamic effect of PLN-R14del, associated with increased mechanical cardiac parameters, such as contractility and fractional shortening, as well as increased Ca^2+^ transients under basal conditions (50). Isoproterenol stimulation did not enhance these parameters, indicating lack of effect by *β*-adrenergic stimulations similarly to the previously reported effect of PKA phosphorylation on SERCA2a Ca^2+^ affinity (5). The mice presented with cardiac remodeling, ventricular dilatation, myocyte disarray and myocardial interstitial fibrosis, in accordance to the overexpression mouse model (50). Intriguingly, in the absence of endogenous PLN, the PLN-R14del mutant protein exhibited a distinct localization pattern as it failed to co-localize with SERCA2a at the SR. Instead, it was found to localize at the plasma membrane, where it interacted with the sarcolemmal Na/K-ATPase, causing its increased enzymatic activity (50). These findings could explain the lack of an effect on SERCA2a that was observed in this PLN-R14del mouse model. Concomitantly, this indicates the detrimental impact of PLN-R14del in homozygous state, which could potentially explain why no homozygous human patients have been reported to date. Although these animal models provided initial insights into PLN-R14del pathology, it should be noted that they are based on overexpression of the PLN-R14del variant (in the wild-type or PLN-KO background) and may thus not fully reflect the human condition.

Advances in experimental protocols have provided alternative gene targeting approaches, enabling the creation of knock-in mouse models that express PLN-R14del at normal levels and may thus be more physiologically relevant to human pathophysiology. Recently, two knock-in mouse models for PLN-R14del have been generated utilizing different experimental strategies that replaced: 1) the endogenous wild type (WT) *Pln* by the mouse mutant *Pln*-R14del sequence, or 2) the endogenous mouse *Pln* by the human *PLN* (WT or R14del) (humanized model) (51, 53, 54). Both mouse models recapitulated human clinical features including contractile dysfunction and increased susceptibility to arrhythmias. In particular, the homozygous model with insertion of the mouse mutant PLN exhibited cardiac dilatation, contractile dysfunction, cardiac fibrosis, high susceptibility to arrhythmias, cardiomyopathy and early mortality (51). Interestingly, heterozygous animals presented delayed onset of cardiomyopathy (occurring after 18 months of age), which could be comparable to human carriers that most often present symptoms at middle age (51). In addition to these cardiac defects, this mouse model presented with PLN aggregates, which were found to occur at early stages of the disease and before the onset of functional deficits (52). Their occurrence is most likely associated with differential expression of genes and proteins involved in protein homeostasis (proteostasis), as indicated by transcriptomics and proteomics analysis (52). With regards to the humanized PLN-R14del mouse model, this has been studied only at the heterozygous state and was shown to exhibit bi-ventricular dilatation, increased stroke volume and marked susceptibility to adrenergic-stimulated ventricular tachycardia at the age of 3–4 months (53). At 12 months, an age that corresponds to when human patients usually present with symptoms, cardiac function was maintained as determined by echocardiography, however, increased fibrosis and lipid droplets were observed in PLN-R14del mouse hearts (55). These features, which are similar to observations in human patients, could serve as substrate for cardiac arrhythmias (41, 42). Moreover, structural abnormalities in Z-disc formation or organization were revealed by transmission electron microscopy and were proposed to contribute to the pro-arrhythmic phenotype of this mouse model (55). At the isolated cardiomyocyte level, detailed analysis of cardiac functional parameters revealed significantly depressed Ca^2+^ kinetics and contractile parameters that were specific to the right ventricle in both young (3–4 months) and older (12 months) mice (54, 55). These changes were similar between male and female mice (55). Importantly, isoproterenol stimulation did not enhance contractile parameters, similar to findings from the PLN-R14del overexpression mouse model where phosphorylation did not fully relieve SERCA2 inhibition (5). Moreover, elevated diastolic Ca^2+^ levels, increased Ca^2+^ leak and Ca^2+^ sparks were determined, which were associated with increased CaMKII activity (54). Consequent aberrations included action potential duration prolongation, increased spontaneous aftercontractions and increased propensity to arrhythmias that originated from the right ventricle (54). Notably, the majority of the observed electrocardiographic alterations were similar to the ones seen in the PLN-R14del patients (54). Detailed electrophysiological studies revealed considerable electrical remodeling that was proposed to contribute towards the initiation of R14del-related arrhythmia (56). In addition to this, decreased maximal force with parallel increased myofilament Ca^2+^ sensitivity were recently reported and were proposed to represent an early compensatory alteration in order to improve contractility in PLN-R14del hearts (55). Importantly, Ca^2+^ homeostasis changes were also corroborated at the gene expression level, with multiple electrophysiological and Ca^2+^ cycling transcripts being altered in PLN-R14del hearts and could contribute to the arrhythmogenic phenotype of these mice (54). In addition, a recent study has uncovered significant splicing alterations that are highly specific to the right ventricle and are related to cardiac action potential regulation (57). These splicing alterations give rise to isoforms associated with arrhythmogenesis and could therefore be directly implicated in PLN-R14del pathogenesis. At the protein level, a recent proteomics analysis revealed expression changes in mitochondrial proteins involved in energy production and metabolism, as well as in proteins associated with myofilament formation and Z-disk organization that were proposed to act as a compensatory mechanism in the remodeling phase of PLN-R14del hearts (53). Additional alterations included protein quality control, as well as desmosomal proteins, which are known to be altered in ACM and could also contribute to PLN-R14del pathology (53).

Apart from these mouse models, a zebrafish model has also recently been described and was created by introduction of the R14del mutation into the zebrafish *plna* gene (58). Adult fish displayed age-related cardiac remodeling and presented sub-epicardial inflammation and fibrosis. In agreement to findings from the knock-in mouse model, homozygous fish displayed severe cardiac morphological changes, which were not observed in heterozygotes (58). Additional pathological features included defects in contractility, action potential alternans and impaired Ca^2+^ homeostasis including increased diastolic Ca^2+^, slower Ca^2+^ transient decay and diminished Ca^2+^ transient amplitude (58). These findings indicate that impaired SR Ca^2+^ sequestration and electrophysiology alterations are underlying PLN-R14del pathophysiology in this zebrafish model, similarly to findings from the humanized mouse model.

Overall, detailed analysis of the numerous animal models has provided insights into the mechanisms underlying PLN-R14del pathophysiology (Figure 1). Observed differences among these models may be due to experimental design (e.g., overexpression vs. knock-in, inclusion of mouse vs. human *PLN* sequence, or expression in WT vs. PLN-KO background), differences in the age that the animals are studied potentially corresponding to various disease progression stages (e.g., pre-symptomatic vs. symptomatic), as well as variations in the experimental procedures and protocols applied.

Collectively, in spite of these variances, currently available PLN-R14del animal models present with pathological features that resemble at varying degrees the human disease. Although none of the available models provides a “perfect” representation of the human phenotype, different aspects of the disease can be investigated in each one. In particular, the knock-in mouse model exhibits late or early phenotype when in heterozygote or homozygote state respectively (51), and could therefore provide means for detailed analysis of the mechanisms associated with disease progression and severity. Moreover, this mouse model is the only one reported so far to present with PLN aggregates, a unique and characteristic feature of PLN-R14del patients (47, 48, 52). Its future in-depth analysis could therefore reveal critical information regarding aggregate formation, as well as possible approaches towards their elimination. On the other hand, detailed characterization of Ca^2+^ handling, cardiac electrophysiology and arrhythmias have been performed in the humanized knock-in mouse model (53, 54, 56). Given that the arrhythmogenic phenotype of this mouse is exacerbated upon stress, it could serve as a suitable model to delineate arrhythmia-triggering mechanisms towards a better understanding of cardiac arrhythmogenesis in human carriers. Based on these key phenotypic features, the different PLN-R14del mouse models, as well as the zebrafish model can provide valuable platforms for evaluation of therapeutic strategies.

## Patient-derived cellular models of PLN-R14del

In parallel to animal models, an increasing number of patient-derived induced pluripotent stem cell cardiomyocyte (iPSC-CM) lines have been created (66–71) (Table 2). Initially, iPSC-CMs were generated from dermal fibroblasts from a female carrier of the original Greek PLN-R14del family (68). In these iPSC-CMs, Ca^2+^ handling abnormalities, electrical instability, abnormal cytoplasmic distribution of PLN-R14del and increased expression of hypertrophic markers were observed (68). Subsequently, defects in Ca^2+^ cycling parameters and contractile dysfunction were reported in other PLN-R14del patient-derived iPSC-CMs (66, 67), as well as in engineered heart tissue (EHT) derived from these iPSC-CMs, albeit at different degrees (67, 72, 73). These aberrations are in agreement to observations in PLN-R14del animal models, therefore implicating their role in disease pathogenesis. However, while experimental evidence from cellular and animal model studies have indicated that PLN-R14del causes depressed SR Ca^2+^ cycling and function, a recent study reported the opposite effect: patient-derived iPSC-CMs did not exhibit any major electrical activity aberrations, but instead appeared to present with accelerated intracellular Ca^2+^ dynamics, findings which led the authors to propose that the mutation may actually result in improved SR Ca^2+^ uptake (66). This is an intriguing finding, potentially in line with enhanced SERCA activity due to partial inhibition by PLN-R14del (5, 74). However, the particular line of iPSC-CMs has been previously extensively characterized and considerable differences in Ca^2+^ transients are observed between the two studies (66, 67). In fact, these iPSC-CMs were previously reported to exhibit Ca^2+^ cycling changes leading to depressed force (67), so it seems somewhat surprising that the authors did not reach similar conclusions. Along those lines, animal model studies (mouse and zebrafish) have determined electrical abnormalities, depressed Ca^2+^ kinetics and impaired Ca^2+^ homeostasis at the cellular and organ level (54, 56, 58), and therapeutic strategies (discussed below) targeting Ca^2+^ scavenging or SERCA activation were reported to have beneficial effects (58, 67, 75). Given that the findings are based on a single patient-derived cell line, further studies will be needed in order to clarify these seemingly contradictory findings.

**Table 2:** PLN-R14del iPSC-CMs and pathogenic features.

iPSC-CM lines	Main pathogenic characteristics	Reference
Karakikes et al.	Ca^2+^ handling abnormalities, electrical instability	(68)
Cuello et al.	Ca^2+^ handling abnormalities, reduced force, alterations of ER/mitochondria	(67)
Feyen et al.	UPR activation	(72)
Badone et al.	Accelerated intracellular Ca^2+^ dynamics	(66)

Apart from Ca^2+^ handling aberrations, in depth analysis of PLN-R14del patient-derived iPSC-CMs have uncovered a range of important molecular alterations associated with this mutation. Specifically, single-cell RNA sequencing in 3 lines of PLN-R14del iPSC-CMs identified increases in endoplasmic reticulum (ER) stress and activation of the unfolded protein response pathway (UPR), an alteration that was also confirmed in patient heart tissue as well as in the humanized PLN-R14del mouse model (54, 72). UPR activation was shown to exert a protective effect and was proposed to act as a compensatory mechanism towards alleviating ER stress and maintaining proteostasis (72). While UPR and ER stress responses were not altered in a different patient-derived line of iPSC-CMs, transcriptomic and proteomic analysis of these iPSC-CMs uncovered aberrations in ER/mitochondrial contact sites as a disease contributing mechanism (67). At the functional level, these changes were associated with post-translational modifications of key metabolic enzymes, perinuclear lipid accumulation, oxidative stress, mitochondrial dysfunction and degeneration. Indeed, transmission electron microscopy of EHTs determined lower abundance of mitochondria in the PLN-R14del samples, which also exhibited large lipid droplets in close association with mitochondria and dilated ER structures. These findings suggest that impairment of the ER/mitochondrial compartment represents another contributing mechanism of disease in PLN-R14del cells (67). Collectively, studies in patient-derived iPSC-CMs have provided critical insights into key aberrant mechanisms associated with PLN-R14del disease and indicate their potential as targets for therapeutic interventions (Figure 1).

## Molecular and biochemical evidence on PLN-R14del defects

Further evidence on the pathological mechanisms of PLN-R14del has been provided by studies performed at biochemical, molecular and structural levels. A number of studies have examined the effect of PLN-R14del on SERCA2a by directly measuring SERCA activity using either heterologous expression in HEK293 cells or reconstituted lipid vesicles with purified proteins (5, 59, 60, 74, 76). All studies agreed that PLN-R14del alone (in the absence of PLN-WT) exhibited inhibitory effects on SERCA activity, albeit less than PLN-WT (67% of the WT according to Ceholski et al.) (74). This is in sharp contrast to other *PLN* mutations, such as PLN-R9C, that result in complete loss of inhibitory function (21, 59, 74), therefore suggesting differences in the respective downstream molecular mechanisms. Since PLN-R14del patients are heterozygotes, when SERCA activity was measured in the presence of both PLN-WT and PLN-R14del proteins, inhibitory effects were either similar to PLN-R14del only samples in assays performed using reconstituted lipid vesicles or enhanced in the case of microsomes from transfected HEK293 cells (5, 60, 74). This discrepancy was proposed to be related to differences in experimental procedures and PLN constructs (60) while the ratio of the proteins used could also be a contributing factor. Nevertheless, it is reasonable to conclude that PLN-R14del exerts some inhibitory effects on SERCA, even in the presence of PLN-WT, but remains to be determined whether this is partial or super-inhibitory. In addition to SERCA activity measurement, a recent study determined significant aberrations in PLN-R14del interactions with an impact on its function. In particular, PLN-R14del was shown to exhibit enhanced association to SERCA2a and HAX-1, probably impacting SERCA2a's Ca^2+^-affinity and function (62). These changes were also confirmed in the humanized PLN-R14del mouse model, demonstrating their *in vivo* relevance in the hearts of this animal model. Additionally, histidine-rich calcium binding protein (HRC) binding to SERCA2a was found to be increased in the presence of PLN-R14del, suggesting enhanced inhibition of SERCA2a maximal velocity. These aberrant SERCA2a interactions possibly underlie the pathological mechanisms, contributing to impaired Ca^2+^ homeostasis and arrhythmogenesis in PLN-R14del hearts (62). Interestingly, PLN-R14del association to SERCA2a remained enhanced even upon PKA phosphorylation, indicating that PLN-R14del binding to SERCA2a is not regulated by phosphorylation. This could be due to inability of PLN-R14del to get phosphorylated at Ser-16 site, as shown both *in vitro* and in transfected cells (59, 60, 62), due to disruption of the PKA motif recognition sequence by the deletion of residue R14 (62). Indeed, significantly weakened PKA interaction (by 10-fold) and reduced phosphorylation kinetics were determined by structural analysis of PLN-R14del in combination with the catalytic subunit of PKA (77, 78). In contrast to this, the CaMKII motif does not appear to be affected by PLN-R14del and thus the mutant protein can still get phosphorylated by CaMKII at the Thr-17 site (61, 62). These findings on PLN-R14del phosphorylation are in agreement to the observed effects on SERCA2a inhibition as well as contractile parameters which are not fully relieved upon isoproterenol stimulation of PLN-R14del cardiomyocytes (5, 54, 76).

Although PLN-R14del was initially proposed to exhibit alterations in its subcellular distribution (50, 68), subsequent findings have not revealed any major changes in its subcellular localization. Immunofluorescence studies in cardiomyocytes from heterozygous mice of the humanized PLN-R14del model revealed a physiological distribution for the PLN-R14del and its co-localization with SERCA2a in both left-ventricular and right-ventricular cardiomyocytes (54). Similarly, in transfected HEK 293 cells, PLN-R14del exhibited ER and perinuclear localization and extensive co-localization to PLN-WT (5, 62), and transfections in the cardiac cell line H9c2 determined that the co-localization of PLN-R14del to SERCA2a is at similar levels as PLN-WT (62). In a recent study on human iPSC-CMs, PLN-R14del was found to co-localize with SERCA2a at both perinuclear and cytosolic regions, however, perinuclear localization of both proteins was found to be reduced (66). This is an interesting finding, especially given the previously described role of PLN in perinuclear Ca^2+^ handling (79, 80), and the potential effects of PLN-R14del along these lines will need to be further explored by future studies.

Another aspect of PLN-R14del mutation that has been investigated, includes possible perturbations in its protein structure. Based on in silico prediction models, PLN-R14del was proposed to exhibit considerable secondary structure changes, including the addition of an extra *α*-helix that results in the disruption of the mutant protein's coil domain (62). This could have significant impact on the protein as the coil domain is believed to form a hinge that connects the two *α*-helical stretches and provides protein flexibility that enables conformational changes, associated with PLN phosphorylation and SERCA2a binding (81). In agreement to the in silico prediction findings, experimental analysis by nuclear magnetic resonance determined perturbations in the helical structure of PLN-R14del cytoplasmic domain, which showed reduced affinity for the phospholipid bilayer surface (60). These structural changes were associated with perturbations in PLN-R14del conformational dynamics and resulted in impaired SERCA2a regulation (76). The findings from these studies demonstrate the critical role of maintaining PLN structure and function in fine-tuning SERCA2a regulation. Along those lines, structural aberrations of PLN-R14del could be directly related to impaired functional properties, including its protein interactions, and could therefore underlie aberrations implicated in disease pathophysiology.

## Existing knowledge of PLN-R14del pathogenetic mechanisms

Extensive research efforts by several groups worldwide have contributed significantly towards our understanding of the mechanisms underlying PLN-R14del pathology. Aberrations in several pathways have been revealed, including protein structural changes, Ca^2+^ handling dysregulation, mitochondrial dysfunction, UPR activation and protein aggregation, each contributing to disease manifestation. Given the functional role of PLN in regulation of Ca^2+^ homeostasis, emphasis has been placed in deciphering defects associated with impaired Ca^2+^ handling, cardiac dysfunction and arrhythmogenesis. Based on current findings, it may be proposed that increased association of PLN-R14del to SERCA2a, most likely due to structural changes of the mutant protein, could affect SERCA2a activity (62). The decreased SR Ca^2+^ uptake and depressed contractility is not relieved by *β*-adrenergic stimulation due to lack of PLN-R14del phosphorylation at the Ser-16 site and mutant PLN becomes a chronic inhibitor of the Ca^2+^-affinity of SERCA2a and Ca^2+^-cycling. At the same time, the enhanced interaction of HRC to SERCA2a in the presence of PLN-R14del may contribute to inhibition of the maximal velocity of SR Ca^2+^ transport and overall increases in diastolic Ca^2+^ levels, promoting a cascade of molecular events and electrophysiological alterations that ultimately result in arrhythmogenesis in PLN R14del hearts (53, 54, 56, 62).

PLN aggregate occurrence, a characteristic feature of PLN-R14del patients, is believed to represent an early trait which is observed before the onset of functional or structural abnormalities in PLN-R14del mouse hearts, therefore possibly implicating a causative role in disease (52). While it is still unknown what triggers PLN aggregate formation, structural alterations of PLN-R14del may cause protein misfolding that could promote its aggregation. The presence of autophagy marker proteins such as p62 and LC3 within PLN aggregates, along with alterations in their expression levels, indicates inefficient protein degradation by the autophagy pathway and depressed proteostasis (48, 82). This defect, along with Ca^2+^ mishandling, may be directly or indirectly implicated in activation of compensatory mechanisms such as UPR in order to alleviate ER stress and maintain proteostasis. In addition, mitochondrial dysfunction could also, at least in part, be related to Ca^2+^ dysregulation, most likely through impaired ER/mitochondrial compartment contacts (67). Consequent defects in mitochondrial function including metabolic dysfunction, oxidative stress and lipid accumulation can also contribute to PLN-R14del disease (46, 52, 53, 67).

Since the majority of these changes are closely interconnected, it is still unclear as to which one represents the initial trigger causing the disease. Aggregate formation was shown to be an early feature, occurring prior to onset of disease in homozygous PLN-R14del mice (52). However, it is not known whether protein structural alterations and/or other pathological characteristics such as Ca^2+^ cycling defects may enhance susceptibility to protein misfolding, thus contributing towards protein aggregation formation. As this mouse model exhibits arrhythmias at early age (6 weeks), even in the heterozygous animals that present with delayed onset of disease (18–20 months), it may be possible to postulate that Ca^2+^ mishandling and electrophysiological defects precede PLN aggregation and could therefore represent processes causal of disease. Further studies are needed to clarify the order of events in this cascade that ultimately leads to PLN-R14del pathogenesis.

## Precision medicine strategies towards targeted PLN-R14del therapy

**Figure 2:**
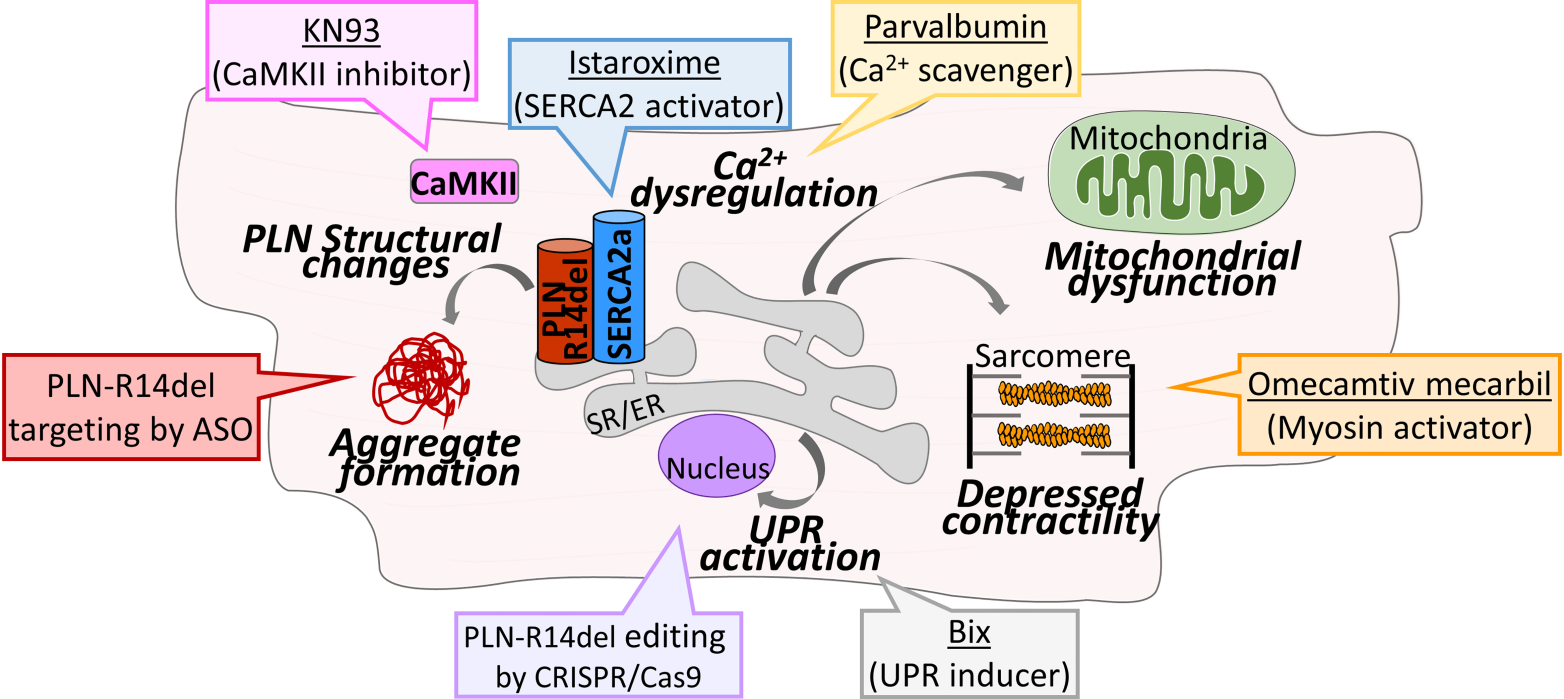
Key pathological mechanisms underlying PLN-R14del and promising strategies for therapeutic interventions. Diagrammatic overview of aberrant molecular mechanisms contributing to PLN-R14del disease pathophysiology, that are currently under investigation as promising targets by different therapeutic approaches (colored boxes).

Following the identification of the mechanisms associated with PLN-R14del pathophysiology, investigation efforts have evaluated possible therapeutic strategies specifically targeted to alleviate PLN-R14del pathogenesis (Figure 2). These have included either gene editing approaches towards correcting the mutant *PLN* sequence as well as molecular and pharmacological strategies that aim to revert key pathological features and prevent molecular cascades leading to disease (Table 3). Gene editing has been successfully performed using transcription activator-like effector nucleases (TALENs) in PLN-R14del iPSC-CMs or clustered regularly interspaced short palindromic repeats (CRISPR) with CRISPR-associated protein 9 (CRISPR-Cas9) in the humanized mouse model (53, 68). Both approaches resulted in correction of *PLN* sequence and significant improvement of PLN-R14del disease phenotypic characteristics. This included rescue of Ca^2+^ cycling and electrophysiological abnormalities, improvement of cardiac function and reduction of arrhythmia susceptibility (53, 68). Pharmacological treatment of iPSC-CMs with the small molecule activator BiP protein Inducer X (BiX) that enhances BiP expression and thus induces UPR was found to have dose-dependent beneficial effects in PLN-R14del iPSC-CM cells (72). In particular, this was associated with amelioration of contractile deficits of PLN-R14del iPSC-CMs and EHTs without affecting Ca^2+^ homeostasis, suggesting its therapeutic potential in preventing contractile dysfunction in PLN-R14del hearts (72). Along those lines, treatment with the myosin activator omecamtiv mecarbil was recently reported to improve contractility of the humanized PLN-R14del cardiomyocytes (55). Another approach exploited the possibility of targeting Ca^2+^ handling dysregulation by the use of Ca^2+^- scavenging proteins such as the Ca^2+^ sensor GCaMP6f or Ca^2+^-binding protein parvalbumin (67). Their application in patient-derived iPSC-CMs resulted in considerable improvement in contractile force, in dynamic changes in ER-mitochondrial compartment, increases in mitochondrial numbers and reduction of specific ER stress/UPR proteins, therefore suggesting the therapeutic potential of targeting Ca^2+^ handling in PLN-R14del (67). Towards this, another approach exploited the potential of enhancing SERCA2a activity in PLN-R14del iPSC-CMs in order to alleviate Ca^2+^ transport dysfunction (75). Indeed, viral expression of a PLN double-mutant (L31A/I40A) that activates SERCA2a function was reported to enhance Ca^2+^ transport and to rescue arrhythmogenic Ca^2+^ transients in PLN-R14del iPSC-CMs, thus suggesting another therapeutic strategy that targets aberrant Ca^2+^ cycling (75). Along those lines, treatment with the small molecule istaroxime that stimulates SERCA2a activity had beneficial effects in the PLN-R14del zebrafish model as it improved cardiac relaxation, ameliorated Ca^2+^ dysregulation by enhancing SR Ca^2+^ uptake and rescued irregular action potential alternans (58). Furthermore, inhibition of CaMKII activity by KN93 was shown to abrogate increased aftercontractions in the humanized mouse model cardiomyocytes, consequently suggesting its anti-arrhythmic application (54) and especially in relation to preventing SCD. Overall, gene editing and pharmacological approaches have rendered promising findings on therapeutic strategies in preventing Ca^2+^ dysregulation, improving contractility and reducing arrhythmia susceptibility in order to alleviate these key pathological features of PLN-R14del pathophysiology.

**Table 3:** Therapeutic approaches for PLN-R14del.

Strategy	Molecular effect	Approach	Model tested	Phenotypic effect	Reference
Gene editing	Gene correction	TALEN	iPSC-CM	Ameliorated Ca^2+^ abnormalities	(68)
	Gene correction	CRISPR-Cas9	Mouse model	Reduced susceptibility to arrhythmias	(53)
	mRNA degradation	ASO	Mouse model	Prevented PLN aggregation	(82, 83)
Pharmacological	UPR inducer	BiX	Mouse model	Ameliorated contractile dysfunction	(72)
	Myosin activator	Omecamtiv mecarbil	Mouse model	Improved contractility	(55)
	Ca^2+^-scavenging	GCaMP6f, Parvalbumin	iPSC-CM	Improved contractile force	(67)
	SERCA2 stimulator	Istaroxime	Zebrafish model	Ameliorated Ca^2+^ dysregulation	(58)
	CaMKII inhibitor	KN93	Mouse model	Prevented aftercontractions	(54)

An alternative gene targeting approach includes the use of antisense oligonucleotides (ASO), which bind to a specific mRNA target resulting in mRNA degradation and enable elimination of a mutant protein without the need for genetic modifications (84). The applicability of this approach in the setting of PLN-R14del pathology was recently investigated in the mouse PLN-R14del knock-in model (82, 83). Administration of a *Pln*-targeting ASO in homozygous PLN-R14del mice prevented PLN aggregation, cardiac remodeling and cardiac dysfunction, leading to a 3-fold increase in survival rate (83). Importantly, beneficial effects were observed even when ASO were administered after disease onset, as ASO treatment prevented disease progression, but could not reverse existing cardiac tissue damage (82). While these are encouraging findings and unveil promising avenues for future studies, it is currently unclear whether such a strategy would actually be applicable in human patients due to inherent differences between mouse and human cardiac physiology, as well as Ca^2+^ cycling mechanisms. In specific, complete loss of PLN in mouse hearts results in enhanced contractile function while in humans it is associated with disease (17, 85). It therefore appears that gene editing approaches aiming to correct PLN-R14del sequence instead of its downregulation may represent more appropriate strategies and along with the latest technological developments could hold promise for human patient therapy (86).

 Collectively, exploitation of multiple approaches has provided promising prospects towards precision medicine strategies for therapeutic interventions in PLN-R14del patients (Figure 2) (49). In addition, the discovery of new therapeutic tools, such as intracellular acting antibodies (intrabodies), may provide additional means that will help combat PLN-R14del pathogenesis and contribute towards the ultimate goal of providing a cure for PLN-R14del patients (87, 88).

## Future perspectives

Despite the tremendous efforts worldwide and the significant scientific progress achieved over the past few years, there are still unanswered questions and issues to be resolved. It is still unclear what factors define and/or influence phenotypic expression of this disease, and why there is such a high phenotypic variability exhibited by PLN-R14del patients. This is expected to be addressed by extensive genotype-phenotype correlation analysis following in-depth clinical and genomic studies that are currently being performed on symptomatic carriers, asymptomatic carriers, as well as non-carrier members from within PLN-R14del families (31, 89). The outcome of this work is anticipated to unveil valuable information on genomic, genetic and/or epigenetic factors influencing disease manifestation. Moreover, by identification of risk factors that predispose to SCD it is envisioned that this cumulative knowledge will ultimately help towards the improved risk stratification of healthy carriers. Accurate patient diagnosis will also be facilitated by the continuously evolving bioinformatical and artificial intelligence tools that expand the available analysis capabilities and open new horizons towards personalized medicine (90–95).

The patient-derived iPSCs and the fabrication of human cardiac tissue and/or organoids from human-derived cells, will be serving as a valuable platform for large scale screening of pharmacological compounds and small molecule libraries, while promising therapeutic approaches could be preclinically tested in the available small and the anticipated large animal models (31). Ultimately, the most promising therapeutic tools will need to be assessed in clinical trials, which are anticipated to progress in a relatively expedited fashion, thanks to the tremendous ongoing work of the PLN Foundation in mapping the PLN-R14del patients/families worldwide.

## Conclusion

Since its discovery in 2006, the PLN-R14del mutation has been attracting an ever increasing research interest worldwide and PLN Foundations organized by the patients have been established in Europe and the US. It remains fascinating how a single amino acid deletion can have such a dramatic effect on PLN function, cells and organs. Aberrations in various pathways have been shown to contribute at varying degrees to disease pathology, while a number of other levels of molecular complexity, such as micro-RNAs, epigenetics and epitranscriptomics, remain to be explored (96–99). While some of the experimental findings may appear conflicting at times, the intense, complementary efforts of the international PLN scientific community ensure continued progress with encouraging prospects towards the identification of promising therapeutic targets. Indeed, multiple therapeutic approaches are yielding increasingly optimistic findings rendering the hope of finding a cure for PLN-R14del patients, a tangible goal.
